# Acoustic Trauma Changes the Parvalbumin-Positive Neurons in Rat Auditory Cortex

**DOI:** 10.1155/2018/9828070

**Published:** 2018-02-08

**Authors:** Congli Liu, Tao Xu, Xiaopeng Liu, Yina Huang, Haitao Wang, Bin Luo, Jingwu Sun

**Affiliations:** ^1^Department of Otolaryngology-Head and Neck Surgery, Anhui Medical University Affiliated Anhui Provincial Hospital, Hefei 230001, China; ^2^Department of Otolaryngology-Head and Neck Surgery, The First Affiliated Hospital of University of Science and Technology of China, Hefei 230001, China; ^3^Department of Otolaryngology-Head and Neck Surgery, Lu'an People's Hospital, Lu'an Affiliated Hospital of Anhui Medical University, Lu'an 237000, China; ^4^Auditory Research Laboratory, University of Science and Technology of China, Hefei 230027, China

## Abstract

Acoustic trauma is being reported to damage the auditory periphery and central system, and the compromised cortical inhibition is involved in auditory disorders, such as hyperacusis and tinnitus. Parvalbumin-containing neurons (PV neurons), a subset of GABAergic neurons, greatly shape and synchronize neural network activities. However, the change of PV neurons following acoustic trauma remains to be elucidated. The present study investigated how auditory cortical PV neurons change following unilateral 1 hour noise exposure (left ear, one octave band noise centered at 16 kHz, 116 dB SPL). Noise exposure elevated the auditory brainstem response threshold of the exposed ear when examined 7 days later. More detectable PV neurons were observed in both sides of the auditory cortex of noise-exposed rats when compared to control. The detectable PV neurons of the left auditory cortex (ipsilateral to the exposed ear) to noise exposure outnumbered those of the right auditory cortex (contralateral to the exposed ear). Quantification of Western blotted bands revealed higher expression level of PV protein in the left cortex. These findings of more active PV neurons in noise-exposed rats suggested that a compensatory mechanism might be initiated to maintain a stable state of the brain.

## 1. Introduction

Acoustic overexposure, aging, and ototoxic drugs could lead to auditory disorders including hearing loss, hyperacusis, and tinnitus [1]. Hearing loss-induced elevation of neuronal activity and synchronization is closely related with impaired inhibition [2–4]. Cortical inhibition was contributed by nearly 20% interneurons which balance the excitation exerted by glutamatergic neurons. These GABAergic interneurons targeting different compartments of glutamatergic neurons play critical roles in sculpturing cortical circuits. GABA inhibition powerfully influences the frequency tuning curve and receptive field of auditory cortex neurons, and the impaired inhibition is implicated in many neurological disorders. Noise-induced increase of the excitability of principal neurons of auditory stations is largely documented [5–7], and now, the question is how the inhibitory neurons change in this process.

Compared with homogenous excitatory neurons, inhibitory neurons are more heterogeneous in terms of morphology, firing patterns, and calcium-binding proteins (CBP) expressed. CBP function as calcium sensor and buffer to regulate calcium signaling and homeostasis [8, 9]. Parvalbumin (PV), calbindin (CB), and calretinin (CR) are characteristic CBP of different subpopulations of interneurons. 20–25% of cortical GABAergic neurons express PV [10], and these neurons belong to fast-spiking interneurons, which play a vital role in the synchrony and oscillation of neural networks. Recently, a layer-specific activity was reported in the noise-induced hearing loss animals as a result of change in cortical GABA neurons [11] and the deterioration of perineuronal nets enwrapping PV neurons [12].

In the present study, immunohistochemical staining and Western blotting assay were applied to quantitate the change of PV inhibitory neurons following chronic acoustic trauma. The findings hopefully advance our understanding of the neural mechanism underlying acoustic trauma-induced hearing loss at the cortical level.

## 2. Materials and Methods

The animal care and experimental procedures were in accordance with the guidelines set by the Institutional Animal Care and Use Committee of Anhui Medical University.

### 2.1. Subjects

31 adult male Sprague-Dawley rats (200–250 g) were randomly divided into two groups, namely control group (8 rats for immunohistochemistry and 7 rats for Western blotting) and noise-exposed group (8 rats for immunohistochemistry and 8 rats for Western blotting).

### 2.2. Auditory Brainstem Evoked Responses

Auditory brainstem evoked responses (ABR) to clicks generated through RZ6 processor (Tucker-Davis Technologies, USA) were obtained (BioSigRZ, Tucker-Davis Technologies, USA) in anesthetized rats (chloral hydrate, 350 mg/kg, i.p.). Three platinum-coated electrodes were placed under the dermis, specifically the positive electrode in the vertex, the ground electrode in the apex nasi, and the negative electrode in the ipsilateral mastoidal dermis. A polyethylene tube of the electrostatic speaker (ED1, TDT) was plugged into the ear canal for sound delivery. Acoustic stimuli were presented at the rate of 10/sec from 100 dB to 5 dB SPL in a descending sequence at 5 dB steps until no discernible waveform was acquired. 1000 repeating stimuli were presented to generate the averaged response. ABR recordings for each ear of each rat were conducted before and on the 7th day after noise exposure.

### 2.3. Unilateral Noise Exposure

Anesthetized rats were unilaterally exposed to one octave band noise centered at 16 kHz with the peak intensity of 116 dB sound pressure level (SPL). The acoustic signal for generating noise was programmed with RpvdsEx v7 (Tucker-Davis Technologies, USA) and MatLab R2008a (MathWorks Inc., USA), generated with TDT System 3 hardware (RP 2.1, PA 5, ED 1, and HB 7), amplified through an amplifier (MATRX/M-640, USA), and presented via a free-field speaker (CP-75A, Shanghai Chuangmu). The noise was converted into electrical signals by a microphone (model 7016, ACO Pacific Inc., USA) and acquired by the TDT system for calibration of sound levels. One hour continuous noise exposure was conducted within a soundproof chamber. The amplified noise was presented via the speaker positioned 3 cm from the left ear canal, while the right ear was carefully plugged to preserve hearing and make a unilaterally noise-exposed animal model. The material of the plug was a kind of propenoic acid, commonly used to make ear mode in clinic, injected into the right external ear canal and ear nail through a syringe; this material could be turned into solid after ten minutes, and it could be easily pulled out from the external ear canal.

### 2.4. Immunohistochemical Staining

Several days after noise exposure, animals were anesthetized to be transcardically perfused with 0.1 M PBS (phosphate buffered saline) and 4% paraformaldehyde fixative. The brains were further postfixed for 6 h at 4°C. 30 *μ*m thick coronal brain slices were cryosectioned with a freezing microtome (CM1950, Leica, Germany). Stereotaxic coordinates [13] were referred to select brain slices containing the auditory cortex. The hippocampus and the rhinal fissure were used as landmarks for locating the auditory cortex. In the coronal slice, we took the edge of the auditory cortex from 1 mm away from the rhinal fissure and we took the width of 1 mm as the auditory cortex. Every fifth section along rostral-caudal axis of the auditory cortex (AC) was collected to form a set of tissue samples. In addition, every one or two of five sets of samples and a total of ten samples of each animal were selected for staining.

Avidin-biotin-peroxidase method (ABC kit, Vector Labs) was adopted to stain PV protein in the 12-well culture plates. Free-floating sections were washed for 10 minutes (3 times) with Tris-Triton (pH 7.4), then incubated for 15 minutes with 10% normal goat serum to block nonspecific sites. Slices were incubated with primary antibody against PV (1 : 1000, PV235, Swant, Switzerland) overnight at 4°C. The secondary antibody (goat anti-mouse IgG, streptavidin-peroxidase kits, ZSJQ-BIO, Beijing, China) was used to biotinylate the primary antibody for 15 minutes, and additional 15 minutes incubation with avidin-biotin-peroxidase solution was performed to form the aggregates. Complete washes with Tris-Triton were done between each incubations. Finally, diaminobenzidine (DAB, ZLI9017, ZSJQ-BIO, Beijing, China) produced the dark brown color reaction to visualize PV neurons, and the sections were further mounted on slides, dehydrated, and coverslipped. The images were taken with a light microscope (ZEISS Axioskop 2 Plus, Germany). The PV neurons across all layers of the auditory cortex were counted with Image Pro Plus 6.0.

### 2.5. Western Blotting Assay

Coronal auditory cortex slices with the thickness of 300 *μ*m were obtained in oxygenated (95% O_2_/5% CO_2_) ice-cold artificial cerebrospinal fluid (ACSF) with a vibratome (DTK-1000, DSK, Japan). ACSF contained the following (in mM): NaCl 129, KCl 3, MgSO_4_ 1.3, KH_2_PO_4_ 1.2, HEPES 3, D-glucose 10, NaHCO_3_ 20, and CaCl_2_ 2.4, with the pH 7.4 and osmolality of 300 Osmol/L. The auditory cortex was carefully dissected out with fine syringe needles from a total of five slices each animal.

Tissues were homogenized manually in a buffer (50 mM Tris, 150 mM NaCl, 0.1% SDS, 1% TritonX-100, and 0.5% sodium deoxycholate, pH 7.6) containing protease inhibitors (Cocktail, Roche, USA). Lysate was cleared at 12000*g* for 10 min at 4°C. The protein concentration of the supernatant was measured through a Bradford assay (Sangon SK3051, Shanghai, China) and quantified through a Biomate5 spectrophotometer (MDC SpectraMax 190, California, USA). 40 mg protein from each sample was added to 5x sample buffer and electrophoresed on 8% sodium dodecyl sulphate-polyacrylamide gel (SDS-PAGE) for 1 h at 120 V. Proteins were transferred from the gel to a 0.45 *μ*m polyvinylidene fluoride- (PVDF-) Plus membrane (Bio-Rad Laboratories Inc., Minnetonka, USA) for 2 h at 260 mA. The target membrane was cut according to marker and blocked at room temperature for 1 h in Tris-buffered saline Tween (TBST, 10 mM Tris/HCl, 150 mM NaCl, 0.1% Tween-20, pH 7.6) containing 5% skim milk and then incubated in TBST containing the primary antibodies at room temperature for 1 h before keeping overnight at 4°C. Following three TBST washes (15 min each), the membrane was incubated in a secondary antibody for 1 h at room temperature. Following another TBST washes, the membrane was developed with an ECL kit (Bio-Rad Laboratories Inc., Minnetonka, USA). Images were acquired using Fusion solo gel imaging system (Vilber Lourmat, France) and were further analyzed using ImageJ (NIH, USA).

The primary antibodies for Western blotting included mouse monoclonal anti-PV (1 : 1000, Swant, Switzerland) and rabbit monoclonal anti-*β*-tubulin (1 : 1000, Cell signaling technology, USA). Secondary antibodies included horseradish peroxidase- (HRP-) conjugated goat anti-mouse and anti-rabbit IgG (1 : 5000, Biosharp, China). The expression level of proteins was quantified with the optic density of a band with ImageJ software, and PV/*β*-tubulin ratio was calculated.

### 2.6. Statistical Analysis

The cell density (cells/mm^2^) was calculated from the PV-positive cells across all auditory cortex layers. SPSS 21.0 (IBM Corporation, Somers, NY) was used for data comparison and presentation. Paired and unpaired Student's *t*-test was taken to evaluate the statistical significance, and difference at the level of *p* < 0.05 was considered significant. All numerical values are expressed as mean ± SE (standard error), and GraphPad Prism software (San Diego, CA, USA) was used for graphs plotting.

## 3. Results

### 3.1. Noise Exposure Elevated ABR Threshold

**Figure 1:**
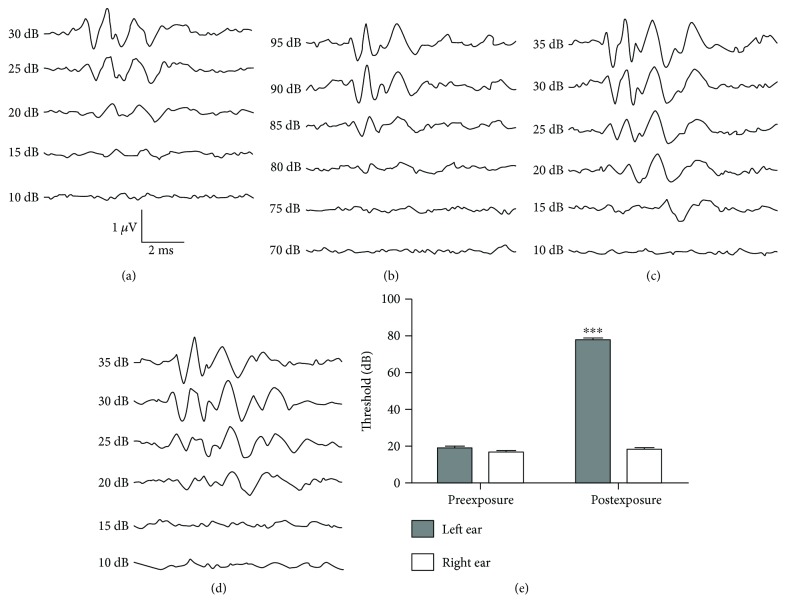
Auditory brainstem responses before and after noise exposure. (a, b) Representative ABR waveforms following acoustic trauma in noise-exposed group ((a) right ear; (b) left ear) and control group ((c) right ear; (d) left ear). (e) Group data showing that ABR threshold was elevated in the exposed ear of exposed group, but not in the unexposed ear and both ears of the control group. ^∗∗∗^*p* < 0.0001.

At first, in order to make sure that all the subjects have a normal hearing before noise exposure, the ABR thresholds for clicks were determined (control group: right ear 19.00 ± 1.01 dB, left ear 18.33 ± 0.79 dB, *n* = 15; exposure group: right ear 18.75 ± 0.85 dB, left ear 17.19 ± 0.91 dB, *n* = 16). The threshold of noise-exposed ear was significantly elevated when the rats were examined 7 days after noise exposure paradigm (76.88 ± 1.01 dB, *p* < 0.0001, *n* = 16, Figure 1(e)), while that of the contralateral ears remained unaffected (18.13 ± 0.77 dB, *p* > 0.05, *n* = 16, Figure 1(e)), which indicated that rat model with unilateral hearing loss was successfully established. The ABR threshold of control group did not show any significant change (data not shown). Representative traces of ABR from each group of rats were shown in Figures 1(a)–1(d).

### 3.2. More Detectable PV Neurons in the Auditory Cortex of Noise-Exposed Rats

**Figure 2:**
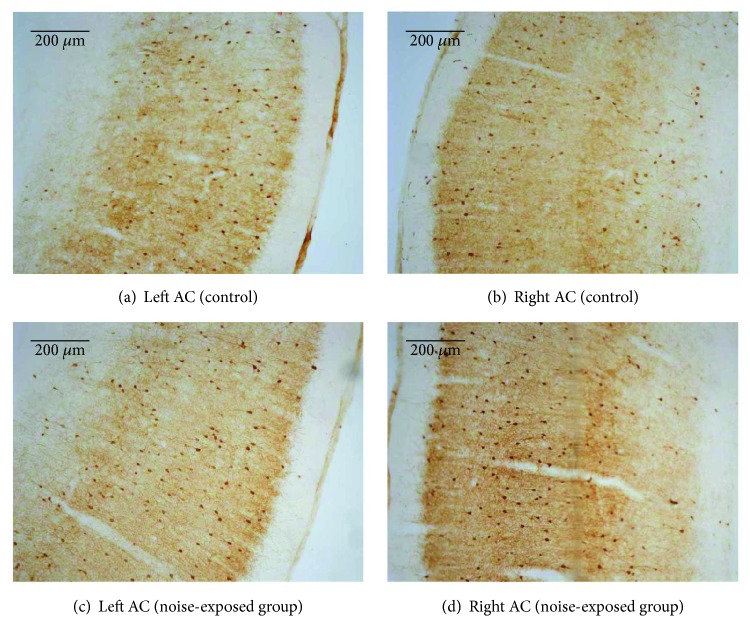
Photographs showing PV-immunoreactive neurons of auditory cortices. Representative immunostaining images of PV in the auditory cortex of a control rat ((a) left AC; (b) right AC) and a noise-exposed rat ((c) left AC; (d) right AC).

**Figure 3:**
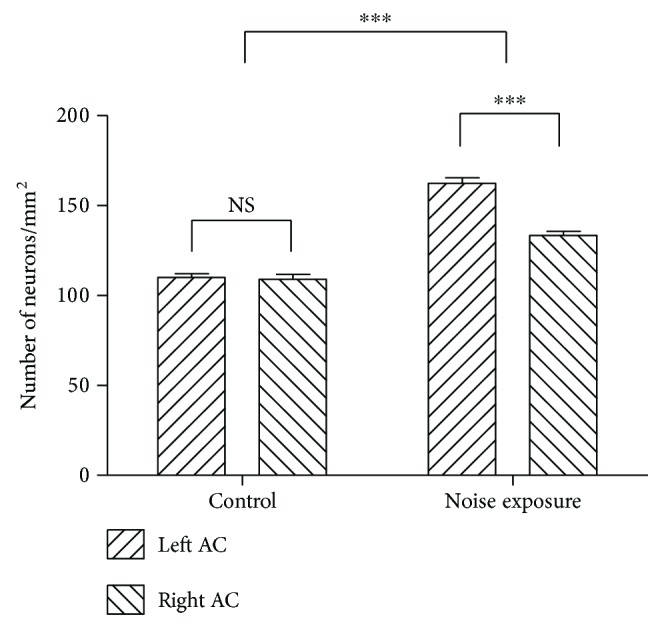
Statistical quantification shows the cell density of detectable PV-positive neurons. Noise exposure elevated the cell density of PV-positive neurons in both sides of the auditory cortex in noise-exposed rats (^∗∗∗^*p* < 0.0001) with higher cell density in the left AC than in the right AC (^∗∗∗^*p* < 0.0001).

As shown in Figure 2, PV-positive neurons are distributed in all cortical layers except layer I. We observed higher density of PV-positive neurons in both sides of the auditory cortex (right AC 133.5 ± 2.21 neurons/mm^2^ and left AC 162.5 ± 2.99 neurons/mm^2^) of noise-exposed rats relative to control group (right AC 109.1 ± 2.77 neurons/mm^2^ and left AC 110 ± 2.05 neurons/mm^2^) (*p* < 0.0001, *n* = 8 for each group, unpaired Student's *t*-test), and the representative photomicrographs for each group and statistical results were shown in Figures 2 and 3, respectively. In the noise-exposed rats, the right AC and left AC are contralateral and ipsilateral side to the exposed ear, and we observed that the number of PV neurons in the left AC exceeds that of the right AC (*p* < 0.0001) after noise exposure.

### 3.3. Noise Exposure Upregulated the Expression Level of Cortical PV

**Figure 4:**
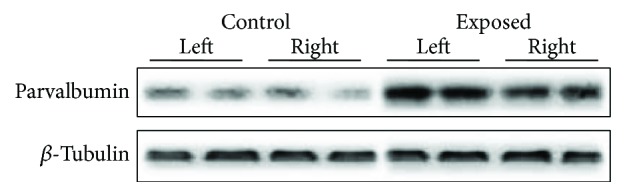
The representative Western blots from a control and a noise-exposed rat.

**Figure 5:**
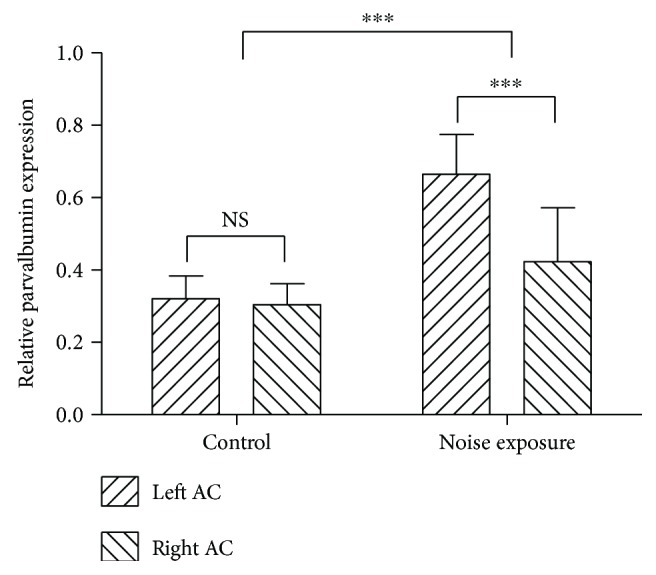
Quantification data showing relative expression levels of parvalbumin in control and noise-exposed rats. ^∗∗∗^*p* < 0.001.

Next, Western blotting was applied to quantify the PV protein level of the auditory cortex before and after noise exposure. On the 7th day following nose exposure, rats were sacrificed for collecting the target tissues, and PV/*β*-tubulin ratio was calculated to indicate the relative expression level of PV. The imaged gel bands from AC of exposed rats were heavier and broader, while those from AC of control rats were lighter and narrower (Figure 4). Statistically, noise exposure significantly upregulated the expression level of cortical PV protein, and the average PV expression level of both sides AC in exposed rats was 174.23% of that in control rats (Figure 5) (*p* < 0.001, *n* = 7 and 8 rats for control and experimental groups, resp.). Comparison between two hemispheres of AC from exposed rats showed that PV expression level of the right AC was 63.64% that of the left AC (*p* < 0.001, *n* = 8).

## 4. Discussion

In the present study, we investigated the effect of acoustic trauma on PV neurons of the auditory cortex, a subset of GABAergic inhibitory neurons. Acoustic trauma, aging, and ototoxic drugs permanently or temporarily produce the hearing deficit. Among these factors, noise exposure becomes more common [14, 15], and noise-induced hearing loss reorganizes the tonotopic maps and elevates the neuronal activity of the auditory cortex, causing other auditory disorders such as tinnitus and hyperacusis [16, 17]. The activity of the brain is influenced by GABAergic inhibition, and the imbalance of excitation and inhibition often occurs following noise exposure [2, 17–20]; hence, it is vital to understand the change of cortical PV neurons following acoustic trauma.

Noise-induced temporary and permanent auditory threshold shifts could be immediately observed depending on the intensity of noise [21–24]. Our noise exposure paradigm caused ABR threshold shift ranging from 45 to 65 dB on day 7 postexposure, which is similar to those reported previously [22, 25, 26]. The underlying mechanism can be acoustic trauma damaging cochlear hair cells [27], and these irreversible insults elevate the auditory threshold [28]. Consistently, the noise-exposed ear with elevated ABR threshold and unexposed ear with normal ABR threshold suggested that the impairment occurred in the ear exposed to noise.

Hearing loss changes the neuronal activities of different auditory stations, and the hypofunction of cortical inhibitory neurons is proposed to account for the overexcitability of auditory principal neurons. PV-containing neurons as the largest population of interneurons target the soma and proximal dendrites of pyramidal neurons to shape the receptive fields [29], process rapid-changing signals [30], and participate in the gamma-band oscillation [31, 32]. Proper function of the brain requires the inhibition mediated by GABA interneurons, and the lowered inhibition is considered to be involved in the hyperactivity of AC and inferior colliculus [33]. Initially, it was hypothesized that noise exposure would decrease the number of PV neurons and protein level of PV, but more PV neurons were detected in both sides of rats' AC. A recent research reported that in mice the cell density of AC PV neurons following bilateral noise exposure indeed showed an increasing trend but having a minor difference [12]. This inconsistence might be a result of various subjects (rats versus mice) and noise exposure paradigm (116 dB unilateral exposure versus 103 dB bilateral exposure).

Quantitative analysis of Western blots of PV protein revealed that acoustic trauma elevated the expression level of PV protein, which is in accordance with more detectable PV neurons in noise-exposed rats. The apparent increase of PV neuron number is unlikely due to the neuronal proliferation in that mammalian adult neurons have already lost their ability of mitosis. The higher protein level and stronger immunoreactivity of PV in AC of noise-exposed rats observed in our experiment and increased evoked-to-spontaneous firing rate ratios in layer II/II PV neurons of AC demonstrated by Novak et al. [11] lead us to propose that a compensatory increase of PV proteins of AC in noise-exposed rats likely makes PV neurons more easily detected. The ipsilateral AC to noise exposure of the noise-exposed rats also underwent a similar change and this could be explained by the fact that binaural information converges on the auditory brain stem and some fibers from peripheral can cross to the contralateral central. One phenomenon which is difficult to explain is why the ipsilateral AC to noise exposure showed a more dramatic change. The surprising finding that the ipsilateral AC to noise exposure showed a more dramatic change could be explained by functional asymmetry between two hemispheres of the auditory cortex. In human being, left AC prevails in processing sound information [34, 35], and in the left ear noise-exposed gerbil [36], the primary AC of the left side was more activated than that of the right side. Similarly, if the activity of the left AC is higher than that of the right AC in our experimental rats, the compensatory mechanism would enable more PV activity in the left AC.

More PV protein expression possibly represents more activated state of cortical neurons, since PV protein, a marker of cellular metabolic level [37, 38], increased in cochlear nucleus and inferior colliculus of mice following noise exposure or sound stimulation [39, 40]. Logically, acoustic trauma-induced change of PV neurons could be suggested to promote cortical inhibition in the noise-exposed rats. However, the percentage of PV neurons of AC superficial layers declined in aged mice AC in a way different from those of noise-induced hearing loss mice [41].

Taken together, this study provided the evidence that acoustic trauma changed the PV neurons expression in rat auditory cortex, and the compensatory change of PV expression would help maintain the balance between excitation and inhibition. Our findings could develop our understanding of the behavior of inhibitory neurons following noise-induced hearing loss and help to develop the prevention or treatment strategies through targeting PV interneurons.
